# Diagnostic accuracy of plasma cell-free DNA qPCR for *Schistosoma haematobium* assessed by Bayesian latent class analysis in a cohort of pregnant women from Lambaréné, Gabon

**DOI:** 10.1186/s40249-026-01447-4

**Published:** 2026-05-09

**Authors:** Youssef Hamway, Theresa Josten, Sherif Abdellatif, Piyas Mukherjee, Julia Schluckebier, Sèyigbéna P. Déo-Gracias Berry, Yabo Josiane Honkpèhedji, Moustapha Nzamba Maloum, Romeo Laclong Lontchi, Paul A. Nguema Moure, Brice Meulah, Pytsje T. Hoekstra, Inge Kroidl, Govert J. van Dam, Andrea Kreidenweiss, Meral Esen, Ayôla Akim Adégnika, Clarissa Prazeres da Costa

**Affiliations:** 1 Institute for Medical Microbiology, Immunology and Hygiene, TUM School of Medicine and Health, Technical University of Munich (TUM), Trogerstrasse 30, 81675 Munich, Germany; 2 Center for Global Health, TUM School of Medicine and Health, Technical University of Munich (TUM), Munich, Germany; 3 German Center for Infection Research (DZIF), Partner Site Munich, Munich, Germany; 4 Institute of Infectious Diseases and Tropical Medicine, LMU University Hospital Munich, Munich, Germany; 5 Centre de Recherches Médicales de Lambaréné (CERMEL), Lambaréné, Gabon; 6 Leiden University Center for Infectious Diseases, Leiden University Medical Centre, Leiden, The Netherlands; 7 Institute for Tropical Medicine, University Hospital Tübingen, Tübingen, Germany; 8 Fondation pour la Recherche Scientifique (FORS), Cotonou, Benin; 9 German Center for Infection Research (DZIF), Partner Site Tübingen, Tübingen, Germany

**Keywords:** Schistosomiasis, Diagnostic accuracy, Molecular diagnostics, Bayesian latent class analysis

## Abstract

**Background:**

Effective diagnosis of *Schistosoma haematobium* is critical for disease management. However, current tools often lack sensitivity for low-intensity infections, a challenge noted in the recent WHO Roadmap for Neglected Tropical Diseases. This study aimed to evaluate a novel cell-free DNA (cfDNA) quantitative Polymerase Chain Reaction (qPCR) assay, utilizing 20 µl of plasma with crude DNA extraction. This approach intends to improve diagnostic accuracy and lower implementation barriers for molecular tests in field settings. We compared its performance against urine filtration microscopy and the Up-converting Reporter Particle Lateral Flow Circulating Anodic Antigen (UCP-LF CAA) test in pregnant women from Lambaréné, Gabon.

**Methods:**

A prospective cross-sectional study was conducted on 296 pregnant women in Gabon. EDTA blood, urine, and stool samples were collected from December 2018 to November 2020. Urine samples were analyzed by urine filtration microscopy and UCP-LF CAA. The cfDNA qPCR assay was performed retrospectively on 20 µl of frozen plasma using a simplified DNA extraction method. Diagnostic accuracy was assessed via sensitivity, specificity, and agreement measures. Bayesian latent class analysis (BLCA) was used to estimate test performance in the absence of a gold standard.

**Results:**

Diagnostic positivity varied: 24.3% by cfDNA qPCR, 18.6% by urine filtration microscopy, and 20.9% by UCP-LF CAA. Agreement between tests was limited. BLCA indicated qPCR to be the most sensitive (74.0%, 95% CrI: 57.2–90.8), followed by UCP-LF CAA (65.7%, 95% CrI: 49.0–82.8). Microscopy was the most specific (94.9%, 95% CrI : 89.6–99.3). Estimated prevalence ranged between 22.5 and 27.1%. Receiver operating characteristic (ROC) analysis confirmed good individual performance (AUC 0.837–0.868), with performance improving when combining any two tests (AUC up to 0.931).

**Conclusions:**

This study validates the high sensitivity of a novel cfDNA qPCR approach using only 20 µl of plasma, demonstrating performance comparable to microscopy and UCP-LF CAA. While microscopy maintains high specificity, combining it with a high-sensitivity test such as qPCR or UCP-LF CAA provides significant diagnostic improvement. Further optimizing the specificity of this streamlined qPCR assay brings us closer to a highly accurate, feasible point-of-care diagnostic crucial for schistosomiasis control programs in resource-limited settings.

**Supplementary Information:**

The online version contains supplementary material available at 10.1186/s40249-026-01447-4.

## Background

Schistosomiasis is a chronic disease caused by parasitic flatworms of the genus *Schistosoma*. It is a disease with a huge global burden with currently more than 250 million people infected worldwide [1]. Being a neglected tropical disease (NTD), most of the affected populations live in rural impoverished areas, with 85% of incidence being in sub-Saharan Africa [2]. As part of the efforts to combat schistosomiasis and the other 21 NTDs, the WHO has conceived a roadmap, with the most recent version outlining the targets for this current decade until 2030 and the actions necessary to achieve them. For schistosomiasis, the target is elimination as a public health problem and to achieve this ambitious goal, action is required in several different sectors from advocacy, capacity building and funding to interventions and novel diagnostics development [3]. Few diagnostics for schistosomiasis already exist and are in routine use. Most commonly these techniques rely on manual egg counting techniques such as urine filtration (*Schistosoma haematobium*) or Kato-Katz smear from fecal samples (*Schistosoma mansoni*), both of which lack sensitivity and throughput [4]. Other tests rely on immunological tests for *Schistosoma* antigens. These include the commercially available point-of-care (POC) CCA test, detecting the circulating cathodic antigen (CCA) released by adult *Schistosoma* worms. This test performs well for the detection of *S. mansoni,* exhibiting higher sensitivity than Kato-Katz [5]. However, it has low sensitivity for the detection of *S. haematobium* [6], as well as reduced specificity in specific populations including pregnant women [7, 8]. Another test also exists for the detection of circulating anodic antigen (CAA), with much improved sensitivity and specificity [9]. However, it is not a POC test nor is it widely available, rather it relies on Up-Converting reporter Particle-Lateral Flow (UCP-LF) technology. A rapid diagnostic test format for the detection of CAA is currently under development [10]. CAA detection was originally developed for the testing of serum samples; however, the use of urine was later introduced to increase ease of sample collection and acceptability [9, 11]. In addition, serology is widely used especially in non-endemic regions to test symptomatic travelers returning from endemic areas or for larger screening purposes [12, 13] whereby this test cannot segregate ongoing from past infections.

Molecular methods have the potential to overcome these limitations, with high throughput, sensitivity and specificity. The go—to molecular testing for infectious disease diagnostics relies on the detection of pathogen DNA via polymerase chain reaction (PCR) and several studies have investigated the applicability of qPCR for the diagnosis of schistosomiasis; however, these mostly rely on egg-rich stool or urine samples, making them only suitable for either *S. mansoni* or *S. haematobium*, but not both [5, 14]. Several studies have also investigated the use of serum for the detection of *Schistosoma* cell-free DNA (cfDNA) [15, 16], however implementation has so far been limited by large sample volume requirements, complex sample preparation steps and the need for equipment that prohibits POC applicability. Other approaches for nucleic acid detection via isothermal amplification such as Loop-mediated isothermal amplification (LAMP) [17, 18] or recombinase polymerase amplification (RPA) [19, 20] have also been explored, though different barriers to their success such as cost and IP protections [21], and technical issues such as difficult assay design and a lack of multiplexing possibilities [17] also exist.

These existing diagnostics are currently deemed insufficient to support the elimination of the disease. Be it due to a lack of sensitivity and specificity, or a lack of POC applicability. As it stands, more sensitive POC diagnostics are needed at all stages from disease mapping to mass drug administration (MDA) decision-making and finally surveillance especially when monitoring elimination progress in particular molecular tests would have the added benefits of supporting xenomonitoring and surveillance, resistance testing as well as testing for other less common manifestations of schistosomiasis such as female genital schistosomiasis (FGS) [22].

Indeed, a well-defined target product profile (TPP) for schistosomiasis diagnostics has been provided recently by a working group at WHO, which details a diagnostic that can detect both *S. mansoni* and *S. haematobium*, being a portable test with a self-contained power source or no power requirements, using a limited sample size of 1–100 µl urine or blood, a throughput of 7–10 individuals per hour with an answer in less than 2 h, a semi-quantitative result and a cost of 1–3 USD/test [23]. The sensitivity and specificity of the ideal test should be at least 60% and 95% respectively, though ideally these would be > 75% and 96.5%. qPCR also possesses qualities that make it ideal for this profile, with a high-throughput and accuracy, though it would require further development to better fit the TPP of a disease such as schistosomiasis, particularly with regards to performance, cost and portability/power requirements.

This study aimed to assess the applicability of a cfDNA qPCR assay for *Schistosoma* detection using a reduced sample volume and processing requirements as an initial assessment towards workflow simplification and future translation to POC molecular platforms. To evaluate the performance of this assay, we first compared it to a composite reference of urine filtration microscopy and UCP-LF CAA. To make this assessment more robust we also conducted a Bayesian latent class analysis, using this to evaluate the sensitivity and specificity of this assay. Using the latent class analysis we also investigated co-variates such as age and co-infections, and their contribution to the likelihood of schistosomiasis in this population.

## Methods

### Study site

The experiments and analyses outlined in this paper are part of a larger study (HelmVit, DRKS00016745) [24] which was conducted at CERMEL (Centre de Recherches Médicales de Lambaréné, Gabon). The aim of the HelmVit study was to assess the impact of schistosomiasis during pregnancy on the Vitamin D metabolism and on the immune system of the offspring. Data and samples were collected from December 2018 to November 2020 in Lambaréné and the surrounding province of Moyen Ogooué in the South-East of Gabon in central Africa, a region characterized by a tropical rainforest climate with high rainfall, extensive river systems, and numerous freshwater bodies that provide suitable habitats for intermediate host snails. These environmental conditions support endemic transmission of *Schistosoma haematobium*, where prevalence is around 23% [25].

### Study population

Pregnant women attending antenatal clinics for routine and/or delivery visits at Hôpital Albert Schweitzer and Centre Hospitalier Régional Georges Rawiri, two health facilities located in the study area, were invited to participate in the study. Participants willing to deliver in the two health facilities were eligible to participate in the study. Participants with known chronic illnesses (diabetes, HIV, hepatitis B/C), were excluded from the study.

### Study design and procedure

The HelmVit study was designed as a prospective cross-sectional study. At baseline, demographics (age, sex and location) and anthropological (weight, height) data were collected. EDTA blood, urine, and stool samples were collected from the participants at the first visit. Plasma was prepared from heparin blood and kept at −80 °C for long-term storage before shipped on dry ice to Munich, Germany, where they were stored until testing in 2024. Only samples from the first visit are included in this evaluation.

### Parasitological examinations and antigen detection

Whole blood was used for the detection of microfilariae by saponin concentration technique, and thick and thin blood smears were made for malaria parasite detection with Giemsa staining and microscopical examination. Urine filtration followed by microscopy was used as the standard diagnosis of urogenital schistosomiasis. In addition, to facilitate a more accurate composite reference standard, CAA was measured in urine samples using the UCP-LF CAA assay. The UCAA*hT*417 dry format of the assay was utilized with a 2 pg/mL threshold [9]. Stool samples were analyzed by direct examination via Kato-Katz, the WHO gold standard for STH egg detection, coproculture and Harada Mori culture techniques for detection of hookworm and *Strongyloides stercoralis* larvae. All examinations were conducted at CERMEL, Lambaréné, Gabon.

### Sample selection

**Fig. 1:**
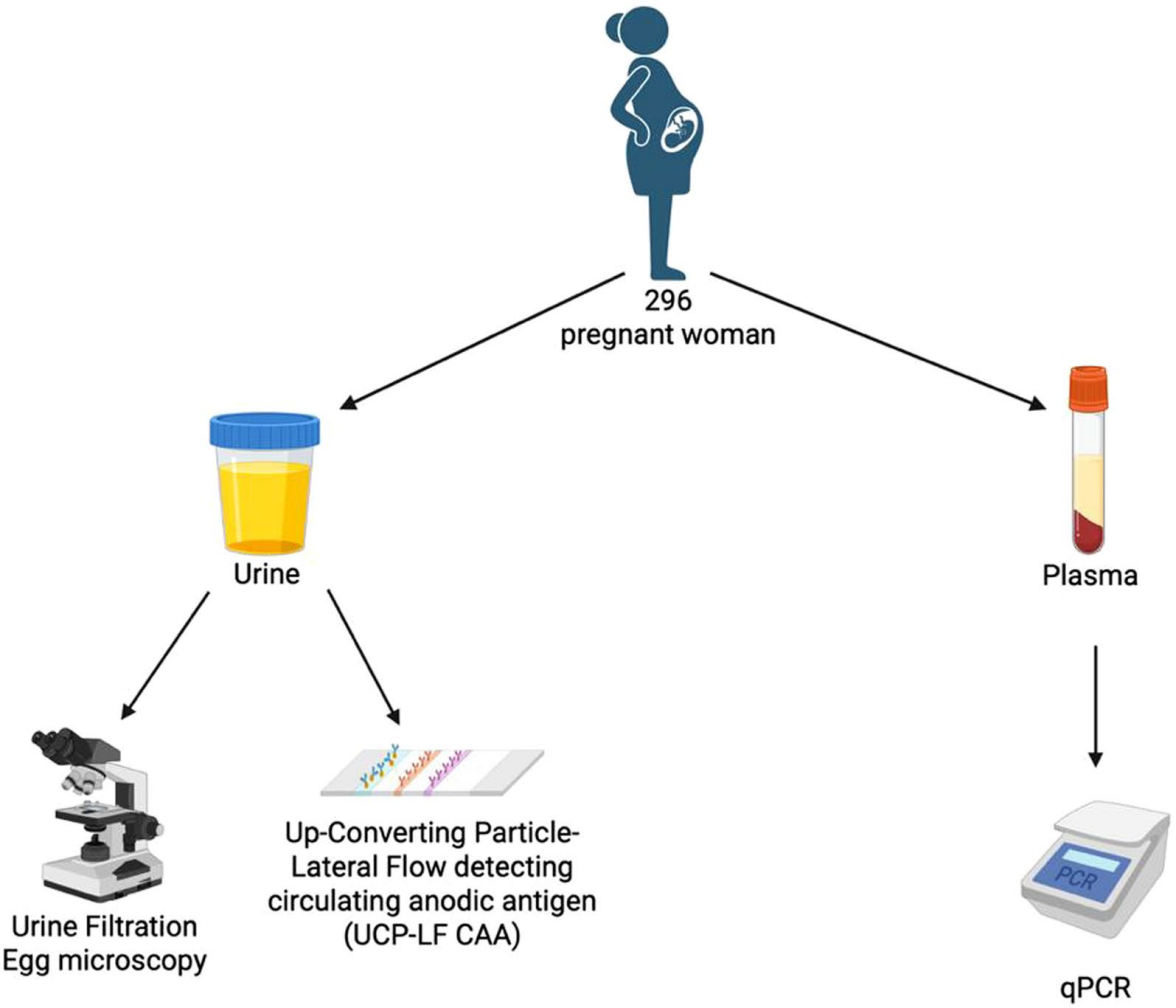
Schematic view of the HelmVit study and diagnostics conducted. The HelmVit study recruited over 600 pregnant women in Lambaréné, Gabon. In the study design urine filtration egg microscopy and UCP-LF CAA were the key diagnostics used to determine *Schistosoma* infection status. All samples were collected at one timepoint. In Munich, 296 of these samples were tested using plasma qPCR. Made with Biorender. qPCR: Quantitative polymerase chain reaction; UCP-LF CAA: Up-converting reporter particle lateral flow circulating anodic antigen

The intended sample size was approximately 300 participants, estimated using standard diagnostic accuracy sample-size formulas [26] based on expected sensitivity of 60%, specificity of 95%, 10% precision, 95% confidence, and an anticipated disease prevalence of ~ 30%. The final dataset included 296 participants, which closely matched the planned sample size. These participants were selected from the original cohort of 648 individuals based on the availability of complete, matched diagnostic data required for comparative analysis. Inclusion required existing results from urine filtration microscopy and UCP-LF CAA testing, as well as sufficient archived plasma volume to perform retrospective cfDNA qPCR analysis (Fig. 1). Participants were excluded due to missing diagnostic results or insufficient plasma volume for molecular testing, rather than predefined clinical or parasitological characteristics.

### DNA isolation and purification for qPCR

DNA was extracted from the plasma samples using the Extracta DNA Prep Kit for PCR (Quantabio, Beverly, Massachusetts, United States) a simple, reagent-based system. This was chosen as a rapid crude extraction approach based on prior use in schistosomiasis molecular diagnostics to enable simplified DNA preparation compatible with resource-limited settings [27], and to evaluate the feasibility of a low-complexity plasma extraction workflow for cfDNA qPCR rather than to maximize cfDNA yield. We added 20 µl of each plasma sample to 100 µl extraction reagent. This was followed by heating to 95 °C for 10 min, then cooling to room temperature. Optionally, we added an equal volume of Stabilization Buffer for extended storage of extracted DNA.

### Primer and probe design

The *S. haematobium* real-time qPCR targeted the Dra1 sequence [GenBank Accession Number: DQ15769 8.1], using the following primers (FW: 5′-GAT CTC ACC TAT CAG ACG AAA CAA AGA-3′; RV: 5′-CGA CCA ACC ATT CCG GAG ATAT-3′) and probe (5′-[6FAM] TGC GAAACAGG CACTTCCAC CAAC [BHQ1]-3′). The amplicon size was 86 bp. The *S. mansoni* real-time qPCR targeted the Sm1-7 sequence [GenBank Accession Number: M61098.1], using the following primers (FW: 5′-GAT CTGA ATC CGA CCA ACC G-3′; RV: 5′-GTT TTA TAT TAA CGC CCA CGC TC-3′) and probe (5′-[6FAM] CTA TGA AAA TCG TTG TAT CTC CGA AAC CAC [BHQ1]-3′). The amplicon size was 115 bp. The *S. mansoni* assay was developed and analytically evaluated in parallel during assay development; however, this assay was not applied to clinical samples in the present study.

### Real-time qPCR

Each qPCR reaction mix (20 μL total volume) contained 4 µmol/L of primers, 14 μL of DNA, 4 µmol/L of probe and 4 μL of AptaTaq Genotyping Master (Roche Diagnostics Deutschland GmbH, Mannheim, Germany). The adjusted protocol included a hot start at 95 °C for 10 min, followed by 45 cycles of 95 °C for 30 s and annealing at 60 °C for 45 s. All qPCR assays were performed on the CFX384 Touch Real-Time PCR Detection System (Bio-Rad, Hercules, California, United States). Analytical sensitivity and species specificity of the qPCR assay were evaluated using serial dilutions of genomic DNA and species-specific controls. Genomic DNA and negative serum samples were included as positive and negative controls on each qPCR plate run, respectively, and no-template controls were included to monitor contamination during PCR setup. No internal amplification or inhibition control was included. No cut-off was implemented as determined by cut-off analysis (data not shown).

### Statistical analysis

All diagnostic variables were harmonized and recorded into binary and continuous formats for analysis, with no missing or indeterminate results. Negative qPCR results were assigned a Ct value of 45.1 to permit inclusion in quantitative analyses. Infection intensity was categorized based on egg count as no eggs, light (1–49 eggs/10 mL), or heavy (≥ 50 eggs/10 mL). Analyses of variability in diagnostic accuracy were exploratory. No pre-specified subgroup analyses were planned; post-hoc comparisons (e.g., by infection intensity and co-infections) were conducted to aid interpretation.

All statistical analyses were performed using R version 4.4.2 (R Foundation for Statistical Computing, Vienna, Austria) within RStudio version 2025.09.2 (Posit Software, Boston, MA, USA). Diagnostic performance metrics including sensitivity, specificity, accuracy and Youden’s J statistic were calculated using the caret, epiR, and DescTools packages in R. Sensitivity and specificity were initially calculated against a UF microscopy and UCP-LF CAA combined reference standard. Diagnostic agreement was visualized using a Venn diagram generated with the euler package. To assess inter-test agreement, Cohen’s kappa was computed for each test pair, and visualized in upper and lower triangle heatmaps, respectively, using the corrplot package.

A Bayesian latent class analysis (BLCA) framework was implemented in R using the *runjags* package to estimate the sensitivity and specificity of urine filtration microscopy, qPCR, and UCP-LF CAA tests in the absence of a gold standard. A binary matrix of observed test outcomes (positive/negative) was passed into the model as input. To assess robustness to model assumptions and prior specification, multiple BLCA model configurations were explored. These included models with and without truncated priors constraining sensitivities and specificities to values ≥ 0.4, introduced as a plausibility constraint to reduce potential non-identifiability and to exclude biologically implausible parameter regions. In addition, models were fitted using either flat (uninformative) priors or informative Beta priors for specificity parameters, based on published diagnostic performance estimates. Informative priors were applied only to specificity, reflecting stronger prior knowledge for specificity than sensitivity across the evaluated tests, while sensitivities were otherwise weakly constrained to allow the data to dominate inference. Informative priors for specificity included Beta(15,5), corresponding to a mean of 75% (95% prior interval approximately 54–91%) for microcopy [28]; Beta(18,3), corresponding to a mean of 86% (95% prior interval approximately 68–97%) for UCP-LF CAA [29]; Beta(9,3), corresponding to a mean of 75% (95% prior interval approximately 48–94%) for qPCR [29]. All models assumed a two-class latent structure (infected vs. uninfected) with a Dirichlet(1,1) prior on class proportions. Posterior distributions were estimated using three parallel chains with a 1,000-sample burn-in and 5,000 retained posterior draws per chain.

Model outputs were compared across configurations based on posterior point estimates and 95% credible intervals of Se and Sp for each test. For model validation and test agreement assessment, we derived individual-level latent class status from the posterior probability of infection and compared these to each test result. Cohen’s kappa coefficients were calculated between LCA-inferred status and observed test outcomes, and receiver operating characteristic (ROC) curves were generated for binary diagnostic results (e.g., PCR Ct, UCP-LF CAA concentration) using the LCA status as the reference. analysis was performed using the pROC package, and AUC values were used to evaluate the discriminative capacity of binary assays individually and in 2-test combinations relative to the latent disease classification.

Pairwise correlations were assessed between qPCR cycle threshold (Ct) values, UCP-LF CAA concentrations, and urine egg counts. For comparability, Ct values were inverted (–Ct) so that higher values indicated greater parasite burden. Spearman’s rank correlation coefficients (ρ) were calculated, with significance determined by two-tailed tests (*P* < 0.05). Associations were visualized with scatterplots and correlation heatmaps using the ggplot2 package in R. All other graphs were made with GraphPad Prism version 10.4.1 (GraphPad software, San Diego, CA, USA).

## Results

### Analytical validation and study cohort

Prior to evaluation in the human cohort, analytical assessment of the qPCR assay targeting DraI (*S. haematobium*) and Sm1.7 (*S. mansoni*) demonstrated a limit of detection of 0.1 pg/µl genomic DNA (Fig. S1) and no cross-reactivity between species targets (data not shown).

To evaluate diagnostic performance, the qPCR assay was assessed in a clinical cohort of pregnant women from Lambaréné, Gabon and compared with established schistosomiasis diagnostics. Here, we focused on a subset of 296 (Fig. 1) participants which had complete diagnostic data for qPCR, urine filtration microscopy and UCP-LF CAA. This cohort consisted exclusively of pregnant women, with a median age of 24 (Table 1).

**Table 1:** Age distribution and diagnostic results of the HelmVit cohort (*N* = 296) from Lambaréné, Gabon (December 2018–November 2020)

Participants	Median age (IQR)	Gestational age at sampling (IQR)
*N* = 296	24 (20–30) years	24 (20–29) weeks

	Positive, *n* (%)	Median value (IQR)
Schistosomiasis diagnostics		
qPCR	72 (24.3%)	36.6 (34.9–38.1)
UCP-LF CAA	62 (20.9%)	11.4 (4.45–30.9)
Urine filtration microscopy	55 (18.6%)	6 (3–21)
STH and other co-infections		
STH	28 (11.8%)	
*Ascaris*	5 (2.1%)	675 (65.25–6007.5)
*Trichuris*	15 (6.3%)	96.5 (39–252.75)
*Hookworm*	8 (3.4%)	128 (80.25–434.7)
Protozoa (*Plasmodium* spp.)	40 (44.9%)	NA
Microfilaria	19 (8.2%)	NA

qPCR: Quantitative polymerase chain reaction; UCP-LF CAA: Up-converting reporter particle lateral flow circulating anodic antigen; STH: Soil-transmitted Helminth; IQR: Interquartile range; NA: Not applicable

### Diagnostic positivity and inter-test agreement

**Fig. 2:**
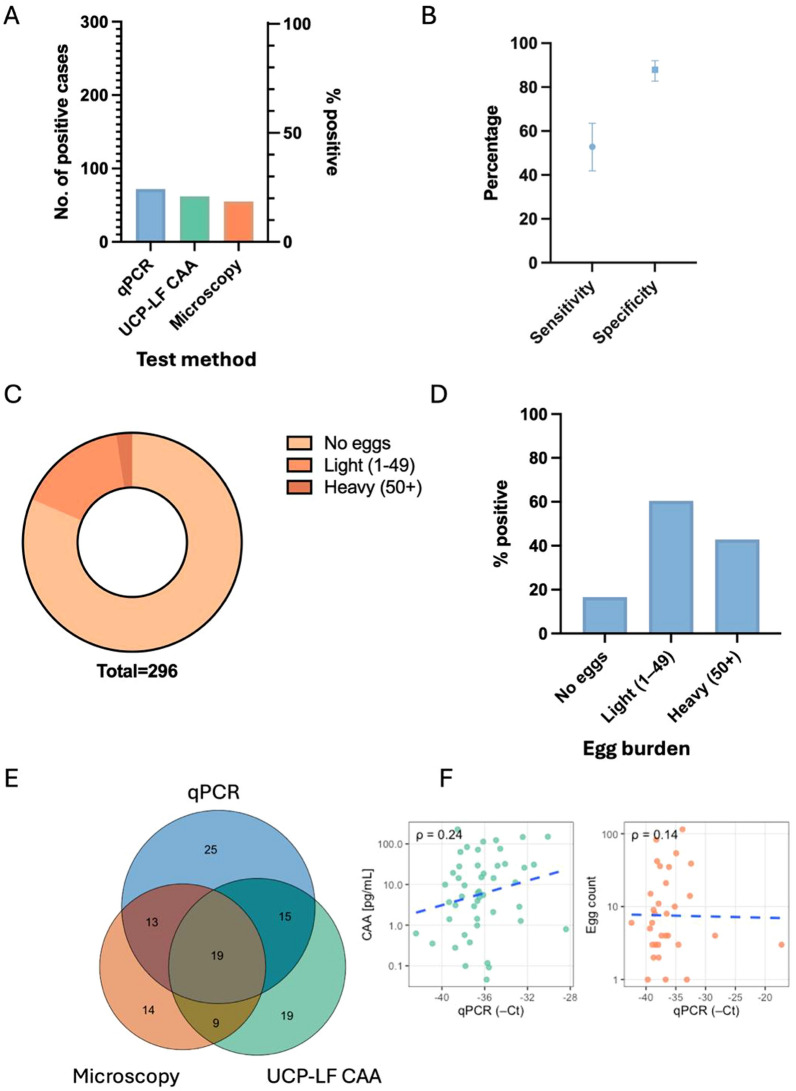
Comparison of diagnostic tests conducted. **A** Positivity rate of all *Schistosoma* diagnostic tests. **B** Sensitivity and specificity (estimate and 95% *CI*) of qPCR as compared to a composite reference standard of urine filtration microscopy and UCP-LF CAA. **C** Proportion of egg microscopy samples with no eggs, light and heavy egg burdens. **D** Percentage of positive cases in each egg burden category, as detected by qPCR. **F** Proportional Venn diagram plot showing no. of individuals positive by the 3 different tests. **E** Pairwise correlation analysis between qPCR and urine filtration microscopy or UCP-LF CAA. Each pairwise correlation is represented in a scatter plot with Spearman’s rho. qPCR: Quantitative polymerase chain reaction; UCP-LF CAA: Up-converting reporter particle lateral flow circulating anodic antigen; *CI*: Confidence interval

First, we looked at the basic positivity rate of each test (Table 1 and Fig. 2a). qPCR identified the most positive cases (24.3%), followed by UCP-LF CAA (20.9%), and then urine filtration microscopy (18.6%). We then used a composite reference standard of urine filtration microscopy and UCP-LF CAA to assess the performance of the qPCR. This resulted in a sensitivity of 52.8% and a specificity of 87.9% for the qPCR (Fig. 2b, Table S1). When calculated against a reference of only urine filtration microscopy the results were similar (58.2% sensitivity, 83.4 specificity) (Fig. S2a, Table S2). 16.6% of microscopy-negative cases were positive by qPCR (Fig. 2d). On the other hand, not all urine microscopy cases with light and heavy egg loads (Fig. 2c) were detected by qPCR (Fig. 2d), highlighting the disagreement between the test methods, evident also in a Venn diagram of all positive cases per diagnostic method (Fig. 2e), where only 19 participants were positive by all 3 tests. This is also reflected in the Cohen’s kappa score (Fig. S2b) where agreement was minimal between the 3 tests (0.35–0.37). In line with the agreement between the binary test results, a weak correlation was also evident when comparing the qPCR Ct values to egg counts or CAA concentration (pg/ml) (Fig. 2f).

### Test performance estimated by Bayesian latent class analysis

**Fig. 3:**
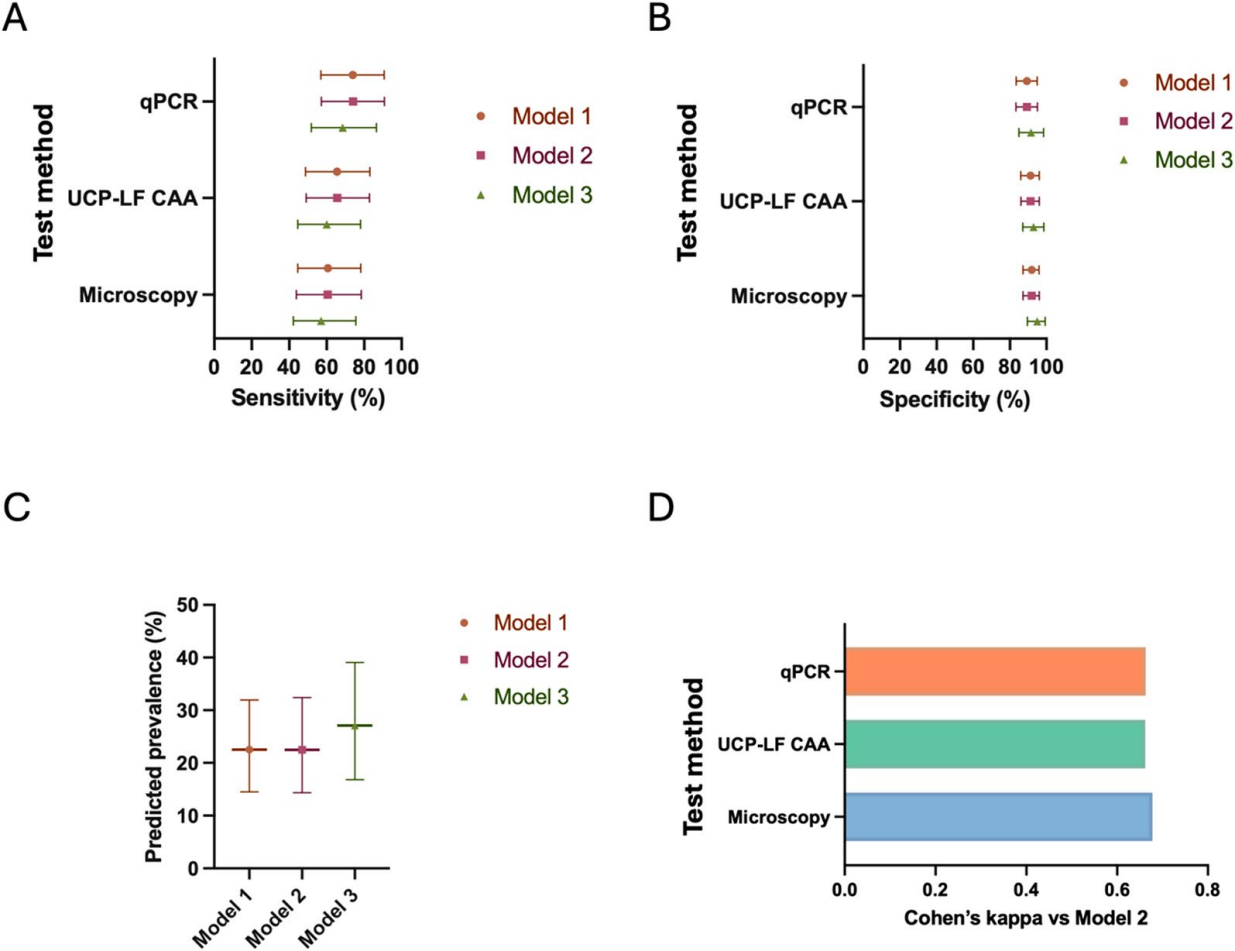
Evaluation of diagnostics performance using BLCA. BLCA models based on microscopy, UCP-LF CAA and qPCR were built using Gibbs sampling (runjags). 3 models were included for diagnostic evaluation using informative priors regarding test specificity (Model 1 and 2) or flat priors (Models 3). Model 1: with truncation. Model 2: no truncation. Model 3: with truncation. **A** Sensitivity and **B** Specificity (estimate and 95% CrI) calculated using each model for qPCR, UCP-LF CAA and microscopy. **C** Prevalence (and 95% CrI) calculated by BLCA Model 2. **D** Cohen’s kappa coefficient between each diagnostic test and BLCA outcome. qPCR: Quantitative polymerase chain reaction; UCP-LF CAA: Up-converting reporter particle lateral flow circulating anodic antigen; BLCA: Bayesian latent class analysis; CrI: Credible interval

**Fig. 4:**
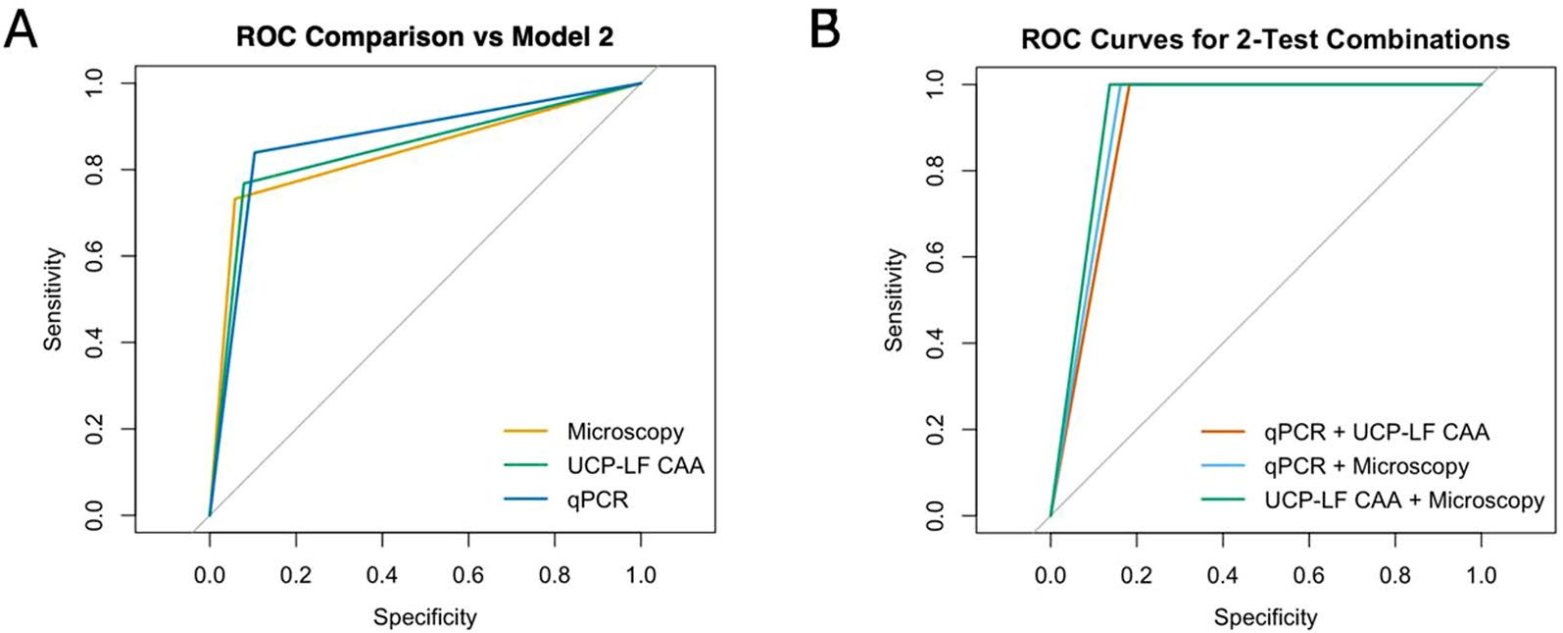
Assessment of individual diagnostic performance vs test combinations. **A** ROC analysis of each diagnostic test vs LCA outcome (Model 2). **B** ROC analysis of test combinations (positive in either test) vs LCA outcome (Model 2)

Next, we calculated sensitivity (Fig. 3a) and specificity (Fig. 3b) of qPCR, urine filtration microscopy and UCP-LF CAA using 3 different BLCA model specifications to assess robustness to prior specification and truncation assumptions. Whereas these were similar among the 3 tests (Table S3), there was a trend across all models tested where qPCR was the most sensitive (68.6–74.0%), followed by UCP-LF CAA (60.0–65.7%) and finally urine filtration microscopy (57.1–60.7%). This order was reversed for specificity (Fig. 3b). The BLCA also estimated prevalence of 22.5–27.1%, depending on the model used (Fig. 3c). Given the stability of estimates across models, Model 2 was selected for subsequent analyses as a simpler, untruncated specification that retained informative priors for specificity. In evaluating which of our diagnostic methods most closely emulated the outcome of the BLCA models we calculated Cohen’s kappa between BLCA Model 2 and each diagnostic test (Fig. 3d). Among the 3 diagnostics, microscopy had the highest agreement (0.68) with the BLCA model, perhaps reflecting the importance of specificity for the accurate diagnosis of schistosomiasis. This was also evident when evaluating the model using a Random Forest classifier (Fig. S3a, b). The classification of individual cases (Fig. S4) also reveals that continuous data from all tests can be used to distinguish BLCA negative vs positive participants. The utility of the 3 diagnostic tests was also reflected in the ROC analysis used to evaluate the sensitivity and specificity of the diagnostic methods as compared to BLCA Model 2 as a reference (Fig. 4a, Table 2). Once again qPCR, UCP-LF CAA and urine filtration microscopy performed similarly (AUC 0.837–0.868); however, combining any two of the tests led to a substantial increase in performance (AUC 0.908–0.931) (Fig. 4b, Table 2).

**Table 2:** Comparison of individual and combined diagnostic approaches as compared to LCA Model 2, calculated using ROC

Test	Sensitivity (%)	Specificity (%)	Accuracy (%)	Youden	AUC
qPCR	83.9	89.6	88.5	0.735	0.868
UCP-LF CAA	76.8	92.1	89.2	0.689	0.844
Microscopy	73.2	94.2	90.2	0.674	0.837
qPCR + UCP-LF CAA	100	81.7	85.1	0.817	0.908
qPCR + Microscopy	100	83.8	86.8	0.837	0.919
UCP-LF CAA + Microscopy	100	86.3	88.9	0.863	0.931

qPCR: Quantitative polymerase chain reaction; UCP-LF CAA: Up-converting reporter particle lateral flow circulating anodic antigen; LCA: Latent class analysis; ROC: Receiver operating characteristic; AUC: Area under the curve

Finally, using our BLCA-defined infection status we assessed whether there is any association between *Schistosoma* infection and other variables such as age or infection with STH or microfilariae. Overall, there was no significant co-variate (Fig. S3c), though trends were evident in terms of increased *Schistosoma* infection in younger woman, and those with other existing infections.

## Discussion

In this study we describe a novel cfDNA qPCR approach for the diagnosis of schistosomiasis using plasma samples which we compared to other tests including urine filtration microscopy and UCP-LF CAA conducted on urine samples. Given the lack of a 100% sensitive reference standard for the diagnosis of *S. haematobium*, and evident disagreement between different test methods, we conducted a Bayesian latent class analysis similar to other recent publications [29–33]. Through this evaluation the cfDNA qPCR performed similarly to urine filtration microscopy and UCP-LF CAA. Although, the cfDNA qPCR was the most sensitive, this was at the cost of specificity. Ultimately, a combination of two tests was found to perform even better. Such an outcome can perhaps be expected, given the tendency to use combined reference standards in academic studies [34–36]. Previous studies have identified UCP-LF CAA and other qPCR approaches as highly sensitive methods for the detection of *S. haematobium* infection [14, 28, 36, 37], however both approaches have suffered from limited implementation due to technical and resource requirements. In this study, we furthermore adapted and improved an existing general DNA extraction method for cfDNA extraction for very low serum or plasma volume of 20 µl, with excellent results, achieving a sensitivity and specificity similar to established methods such as urine filtration microscopy and antigen detection methods such as UCP-LF CAA. This approach strives towards the WHO TPP goal of diagnosis using a finger-prick blood drop, though this requires further development. Even though the volume used is within the correct range [38], we have not yet tested extraction from finger-prick blood directly. Furthermore, this method does not require a centrifuge and is otherwise cost-effective (~ 1 USD per sample), therefore reducing some of the technical and economic barriers to qPCR. The additional reduction of sample input from the 500 µl–1 ml usually seen in qPCR studies [39–42] highlights the sensitivity of this assay and further aids implementation in existing studies. Overall, whereas new sensitive, rapid and field applicable diagnostics remain a priority, existing diagnostics used in combination could be a valid approach. Additionally, refinement of sample processing can leverage the capabilities of existing diagnostic approaches and align them further with TPP goals, this includes improvement in sample input volumes, processing time and equipment needs.

Although PCR-based detection of *S. haematobium* has been widely evaluated, only a limited number of studies have assessed this using serum/plasma cfDNA. In imported schistosomiasis cohorts, Guegan et al. reported a duplex serum qPCR with overall 72.7% sensitivity and 98.9% specificity [43]. Meanwhile, in a similar cohort Frickmann et al. estimated serum duplex real-time PCR performance of 94.9% sensitivity and 98.4% specificity using an LCA [44]. In endemic settings, Lorenz et al. reported serum-based detection of species-specific cfDNA in rural Madagascar, with BLCA-derived diagnostic accuracy estimates of 95.2% sensitivity and 60.3% specificity [29]. Ouédraogo et al. reported LCA estimates for *S. haematobium* plasma cfDNA of 76% sensitivity and 97% specificity in Burkina Faso [45]. In the present study, the cfDNA qPCR assay demonstrated BLCA-derived sensitivity estimates of 68.6–74.0% with specificity of 89.3–91.6% across model specifications, which is broadly consistent with the plasma cfDNA performance reported in endemic populations, while showing lower accuracy than values reported in some imported-disease serum PCR cohorts, reflecting important differences that can arise due in variation in study and transmission settings.

It is also important to expand on the lack of agreement between the 3 test methods evaluated. These differences may be partly explained by the differences in sample type (urine vs. plasma), the analyte (cfDNA, CAA, eggs), and its potential biological origin (adult worms, juvenile worms, eggs). However, ultimately a test that can identify infection regardless of infection stage or sample type limitations is urgently needed, here the versatile utility of qPCR across sample types and infection stages can be a potential solution.

Samples used in this investigation were derived from a field study investigating schistosomiasis and other infections during pregnancy and their effects on the immune system of the newborn (HelmVit study) [46], which was conducted in Lambaréné, Gabon. Here, the estimated and previously reported prevalence is about 23% [47, 48], which is in line with our BLCA estimates of 22.5–27.1% (depending on the model). Few studies assessing diagnostic accuracy have been in conducted in Gabonese populations, recently published studies include a study in Lambaréné evaluating the Schistoscope [49], an AI-based microscope which exhibited somewhat better sensitivity than conventional urine filtration microscopy. Another study conducted in south-east Gabon identified 99.0% sensitivity and 93.3% specificity for urine PCR (Dra1), outperforming microscopy which had a sensitivity of 74.8%, specificity of 93.3% [50]. Of note here is the use of urine samples for the PCR, which has been shown to have high sensitivity, though it can only be used to detect *S. haematobium* and would not fulfil WHO TPP goals of a sample input volume < 100 µl [23].

Another important difference in our study is the focus on pregnant women, a key group often underrepresented in schistosomiasis research despite being particularly vulnerable, with few studies assessing diagnostic performance in this group. Studies have already described a reduced egg burden in women as compared to men [51], whereas others have highlighted suppressed immune responses in pregnant women [52], which may affect egg excretion thus potentially requiring more sensitive detection methods. In addition, pregnancy-associated plasma volume expansion and hemodilution could plausibly reduce circulating concentrations of parasite-derived biomarkers (including cfDNA) [53], particularly in low-intensity infections. These physiological changes may therefore influence the sensitivity of blood-based diagnostics independently of parasite burden. Although our study was not powered to formally quantify pregnancy-specific effects on diagnostic performance, this highlights the need for dedicated comparisons between pregnant and non-pregnant women (and men) using robust reference frameworks. Of note here is the freeBILy study [54, 55], also conducted in Lambaréné, Gabon, which is specifically evaluating UCP-LF CAA accuracy in pregnant women as compared to other diagnostic methods, with preliminary findings suggesting a sensitivity of 69% for UCP-LF CAA as compared to a composite reference standard of urine qPCR, UCP-LF CAA and urine filtration microscopy, higher than urine filtration microscopy (59%) and urine qPCR (47%) (Honkpèhedji et al. manuscript in preparation). Aside from the PCR these findings are similar to those observed in this study; furthering highlighting the need for continued optimization of the sensitivity and specificity of molecular methods. In general, schistosomiasis in pregnancy has been linked to maternal anemia, low birth weight and increased morbidity for both mother and child [56–58]. Therefore, adoption of more sensitive diagnostics could prompt earlier treatment, potentially improving maternal health and fetal growth outcomes.

### Limitations

The main limitation of this study is that the population consisted exclusively of pregnant women, which may limit the generalizability of the diagnostic accuracy estimates to other demographic groups. Although some previous studies have reported reduced performance of standard diagnostics such as urine filtration microscopy in pregnancy, the same study found qPCR performance to be robust, suggesting this may not substantially bias our findings [59]. A second limitation is that the analysis only included participants with complete matched diagnostic data, which may introduce selection bias. Exclusions were driven by logistical factors, including missing diagnostic results or insufficient archived plasma volume for retrospective cfDNA qPCR analysis, rather than predefined clinical or parasitological characteristics. As a result, substantial bias in comparative test performance is considered unlikely; however, some impact on prevalence estimates and sensitivity cannot be fully excluded. A further limitation relates to the use of BLCA in the absence of a true gold standard. Although BLCA mitigates the limitations of imperfect reference tests, it relies on the same diagnostic assays under evaluation to infer latent infection status, introducing an element of circularity. As a result, sensitivity and specificity estimates are not fully independent of the evaluated tests and may be influenced by shared information or residual dependencies, which can affect absolute accuracy estimates. In addition, BLCA results depend on assumptions regarding model structure and prior specification, and while we explored multiple model configurations to assess robustness; some influence of these assumptions, particularly on sensitivity estimates, cannot be fully excluded. Finally, the cfDNA qPCR assay used only 20 µl of plasma, which could theoretically underestimate sensitivity compared with higher-volume approaches. However, our internal evaluations (data not shown) did not indicate a loss of performance, and the strong diagnostic accuracy observed in this study further supports the robustness of the assay. Although this simplified workflow performed well in the present evaluation, the extraction method used has not been formally validated for plasma cfDNA recovery, and suboptimal recovery of highly fragmented DNA cannot be fully excluded; further cfDNA-specific validation and optimization may improve diagnostic performance. Furthermore, the absence of a formal internal amplification control in the present study means that PCR inhibition or extraction failure cannot be completely excluded, particularly in samples with low target concentrations, which may increase false negatives and thereby underestimate qPCR sensitivity.

## Conclusions

In this study, we have evaluated the performance of a novel cfDNA qPCR based on 20 µl of plasma through comparison to urine filtration microscopy and UCP-LF CAA using BLCA. Through this evaluation we identified robust performance of the qPCR assay performing similarly to the other established methods, with higher sensitivity. In particular, combining a higher sensitivity test (UCP-LF CAA, qPCR) with a higher specificity test (urine filtration microscopy), yielded even better outcomes as expected, but at the expense of increased complexity and resource requirements. Together these findings suggest that further refinement of the cfDNA qPCR assay specificity, as well as validation of DNA extraction and sample processing methods for finger-prick blood, and other samples of interest e.g. cervicovaginal lavage or swabs for FGS, can enable steps towards translation to decentralized or POC molecular platforms.

## Supplementary Information

Supplementary material 1.

## Data Availability

The datasets used and/or analyzed during the current study are available from the corresponding author on reasonable request.

## Abbreviations

AUC: Area under the curve
BLCA: Bayesian latent class analysis
CAA: Circulating Anodic Antigen
(UCP-LF) CAA: (Up-converting reporter particle lateral flow) circulating anodic antigen
CCA: Circulating Cathodic Antigen
cfDNA: Cell-free DNA
*CI*: Confidence interval
CrI: Credible interval
Ct: Cycle threshold
DZIF: German Center for Infection Research
ELISA: Enzyme-linked immunosorbent assay
FGS: Female genital schistosomiasis
LAMP: Loop-mediated isothermal amplification
LCA: Latent class analysis
MDA: Mass Drug Administration
NTD: Neglected tropical disease
PCR: Polymerase chain reaction
POC: Point-of-care
POC-CCA: Point-of-care circulating cathodic antigen
qPCR: Quantitative polymerase chain reaction
ROC: Receiver operating characteristic
RPA: Recombinase polymerase amplification
Se/Sp: Sensitivity/Specificity
STH: Soil-Transmitted Helminth
TPP: Target product profile
UCP-LF: Up-converting reporter particle–lateral flow
UF: Urine filtration
WHO: World Health Organization

## References

[1] LoVerde PT. Schistosomiasis. Adv Exp Med Biol. 2024;1454:75–105. 39008264 10.1007/978-3-031-60121-7_3

[2] Aula OP, McManus DP, Jones MK, Gordon CA. Schistosomiasis with a focus on Africa. Trop Med Infect Dis. 2021;6:109. 34206495 10.3390/tropicalmed6030109 PMC8293433

[3] Casulli A. New global targets for NTDs in the WHO roadmap 2021–2030. PLoS Negl Trop Dis. 2021;15:e0009373. 33983940 10.1371/journal.pntd.0009373 PMC8118239

[4] Chala B. Advances in diagnosis of schistosomiasis: focus on challenges and future approaches. Int J Gen Med. 2023;16:983–95. 36967838 10.2147/IJGM.S391017 PMC10032164

[5] Alemu G, Nibret E. Evaluation of the urine POC-CCA test accuracy in the detection of Schistosoma mansoni infection: a systematic review and meta-analysis. J Trop Med. 2024;2024:5531687. 39040853 10.1155/2024/5531687 PMC11262874

[6] Sanneh B, Joof E, Sanyang AM, Renneker K, Camara Y, Sey AP, et al. Field evaluation of a schistosome circulating cathodic antigen rapid test kit at point-of-care for mapping of schistosomiasis endemic districts in The Gambia. PLoS ONE. 2017;12:e0182003. 28797128 10.1371/journal.pone.0182003 PMC5552248

[7] Marti H, Halbeisen S, Bausch K, Nickel B, Neumayr A. Specificity of the POC-CCA urine test for diagnosing S. mansoni schistosomiasis. Travel Med Infect Dis. 2020;33:101473. 31505266 10.1016/j.tmaid.2019.101473

[8] Casacuberta-Partal M, Beenakker M, de Dood CJ, Hoekstra PT, Kroon L, Kornelis D, et al. Specificity of the point-of-care urine strip test for schistosoma circulating cathodic antigen (POC-CCA) tested in non-endemic pregnant women and young children. Am J Trop Med Hyg. 2021;104:1412–7. 33534739 10.4269/ajtmh.20-1168 PMC8045634

[9] Corstjens P, de Dood CJ, Knopp S, Clements MN, Ortu G, Umulisa I, et al. Circulating Anodic Antigen (CAA): a highly sensitive diagnostic biomarker to detect active schistosoma infections-improvement and use during SCORE. Am J Trop Med Hyg. 2020;103:50–7. 32400344 10.4269/ajtmh.19-0819 PMC7351307

[10] FIND. Rapid test for precision mapping, and monitoring and evaluation of schistosomiasis control programmes. 2025. https://www.finddx.org/what-we-do/projects/rapid-test-for-precision-mapping-and-monitoring-and-evaluation-of-schistosomiasis-control-programmes/. Accessed 11 Sept 2025.

[11] Corstjens PL, Nyakundi RK, de Dood CJ, Kariuki TM, Ochola EA, Karanja DM, et al. Improved sensitivity of the urine CAA lateral-flow assay for diagnosing active Schistosoma infections by using larger sample volumes. Parasit Vectors. 2015;8:241. 25896512 10.1186/s13071-015-0857-7 PMC4418045

[12] Grzegorek K, Kroidl I, da Costa CP, Rothe C. Spectrum of helminth infections in migrants from Sub-Saharan Africa to Europe: a literature review. Am J Trop Med Hyg. 2023;108:1096–104. 37094791 10.4269/ajtmh.22-0354 PMC10540113

[13] Hinz R, Schwarz NG, Hahn A, Frickmann H. Serological approaches for the diagnosis of schistosomiasis - a review. Mol Cell Probes. 2017;31:2–21. 27986555 10.1016/j.mcp.2016.12.003

[14] Keller D, Rothen J, Dangy JP, Saner C, Daubenberger C, Allan F, et al. Performance of a real-time PCR approach for diagnosing Schistosoma haematobium infections of different intensity in urine samples from Zanzibar. Infect Dis Poverty. 2020;9:128. 32887642 10.1186/s40249-020-00726-y PMC7487541

[15] Cnops L, Tannich E, Polman K, Clerinx J, Van Esbroeck M. Schistosoma real-time PCR as diagnostic tool for international travellers and migrants. Trop Med Int Health. 2012;17:1208–16. 22882536 10.1111/j.1365-3156.2012.03060.x

[16] Fuss A, Mazigo HD, Mueller A. Detection of Schistosoma mansoni DNA using polymerase chain reaction from serum and dried blood spot card samples of an adult population in North-western Tanzania. Infect Dis Poverty. 2021;10:15. 33622417 10.1186/s40249-021-00798-4 PMC7901113

[17] Garcia-Bernalt Diego J, Fernandez-Soto P, Febrer-Sendra B, Crego-Vicente B, Muro A. Loop-mediated isothermal amplification in schistosomiasis. J Clin Med. 2021;10:511. 33535489 10.3390/jcm10030511 PMC7867102

[18] Mesquita SG, Neves F, Scholte RGC, Carvalho ODS, Fonseca CT, Caldeira RL. A loop-mediated isothermal amplification assay for Schistosoma mansoni detection in Biomphalaria spp. from schistosomiasis-endemic areas in Minas Gerais, Brazil. Parasit Vectors. 2021;14:388. 34362440 10.1186/s13071-021-04888-y PMC8343921

[19] Mesquita SG, Gadd G, Coelho FS, Cieplinski A, Emery A, Lugli EB, et al. Laboratory and field validation of the recombinase polymerase amplification assay targeting the Schistosoma mansoni mitochondrial minisatellite region (SmMIT-RPA) for snail xenomonitoring for schistosomiasis. Int J Parasitol. 2024;54:247–56. 38311021 10.1016/j.ijpara.2024.01.005

[20] Rostron P, Pennance T, Bakar F, Rollinson D, Knopp S, Allan F, et al. Development of a recombinase polymerase amplification (RPA) fluorescence assay for the detection of Schistosoma haematobium. Parasit Vectors. 2019;12:514. 31685024 10.1186/s13071-019-3755-6 PMC6827214

[21] Tan M, Liao C, Liang L, Yi X, Zhou Z, Wei G. Recent advances in recombinase polymerase amplification: Principle, advantages, disadvantages and applications. Front Cell Infect Microbiol. 2022;12:1019071. 36519130 10.3389/fcimb.2022.1019071 PMC9742450

[22] World Health Organization. Ending the neglect to attain the Sustainable Development Goals: a road map for neglected tropical diseases 2021–2030. Geneva: World Health Organization; 2020.

[23] World Health Organization. Diagnostic target product profiles for monitoring, evaluation and surveillance of schistosomiasis control programs. Geneva: World Health Organization; 2021. Report No.: 9789240031104.

[24] Centre de Recherche Médicales de Lambaréné. Helminth infection during pregnancy on vitamin D regulation: HELMVIT study. 2023. https://ctv.veeva.com/study/helminth-infection-during-pregnancy-on-vitamin-d-regulation-helmvit-study?utm. Accessed 11 Sept 2025.

[25] Dejon-Agobe JC, Honkpehedji YJ, Zinsou JF, Edoa JR, Adegbite BR, Mangaboula A, et al. Epidemiology of Schistosomiasis and Soil-Transmitted Helminth Coinfections among Schoolchildren Living in Lambarene, Gabon. Am J Trop Med Hyg. 2020;103:325–33. 32431272 10.4269/ajtmh.19-0835 PMC7356410

[26] Buderer NM. Statistical methodology: I. Incorporating the prevalence of disease into the sample size calculation for sensitivity and specificity. Acad Emerg Med. 1996;3:895–900. 8870764 10.1111/j.1553-2712.1996.tb03538.x

[27] Archer J, Patwary FK, Sturt AS, Webb EL, Phiri CR, Mweene T, et al. Validation of the isothermal Schistosoma haematobium Recombinase Polymerase Amplification (RPA) assay, coupled with simplified sample preparation, for diagnosing female genital schistosomiasis using cervicovaginal lavage and vaginal self-swab samples. PLoS Negl Trop Dis. 2022;16:e0010276. 35286336 10.1371/journal.pntd.0010276 PMC8947142

[28] Knopp S, Corstjens PL, Koukounari A, Cercamondi CI, Ame SM, Ali SM, et al. Sensitivity and specificity of a urine circulating anodic antigen test for the diagnosis of Schistosoma haematobium in low endemic settings. PLoS Negl Trop Dis. 2015;9:e0003752. 25973845 10.1371/journal.pntd.0003752 PMC4431728

[29] Lorenz E, Razafindrakoto R, Rausche P, Rasolojaona ZT, Razafindralava NM, Zerbo A, et al. Detecting Schistosoma infections in endemic countries: a diagnostic accuracy study in rural Madagascar. Infect Dis Poverty. 2025;14:20. 40098012 10.1186/s40249-025-01292-x PMC11912594

[30] Calvo-Urbano B, Leger E, Gabain I, De Dood CJ, Diouf ND, Borlase A, et al. Sensitivity and specificity of human point-of-care circulating cathodic antigen (POC-CCA) test in African livestock for rapid diagnosis of schistosomiasis: a Bayesian latent class analysis. PLoS Negl Trop Dis. 2023;17:e0010739. 37216407 10.1371/journal.pntd.0010739 PMC10237635

[31] Clark J, Moses A, Nankasi A, Faust CL, Adriko M, Ajambo D, et al. Translating from egg- to antigen-based indicators for Schistosoma mansoni elimination targets: a Bayesian latent class analysis study. Front Trop Dis. 2022;3:825721. 35784267 10.3389/fitd.2022.825721 PMC7612949

[32] Clements MN, Corstjens P, Binder S, Campbell CH Jr, de Dood CJ, Fenwick A, et al. Latent class analysis to evaluate performance of point-of-care CCA for low-intensity Schistosoma mansoni infections in Burundi. Parasit Vectors. 2018;11:111. 29475457 10.1186/s13071-018-2700-4 PMC5824563

[33] Mesquita SG, Caldeira RL, Favre TC, Massara CL, Beck L, Simoes TC, et al. Assessment of the accuracy of 11 different diagnostic tests for the detection of Schistosomiasis mansoni in individuals from a Brazilian area of low endemicity using latent class analysis. Front Microbiol. 2022;13:1048457. 36590409 10.3389/fmicb.2022.1048457 PMC9797737

[34] Lodh N, Naples JM, Bosompem KM, Quartey J, Shiff CJ. Detection of parasite-specific DNA in urine sediment obtained by filtration differentiates between single and mixed infections of Schistosoma mansoni and S. haematobium from endemic areas in Ghana. PLoS One. 2014;9:e91144. 24632992 10.1371/journal.pone.0091144 PMC3954594

[35] Midzi N, Butterworth AE, Mduluza T, Munyati S, Deelder AM, van Dam GJ. Use of circulating cathodic antigen strips for the diagnosis of urinary schistosomiasis. Trans R Soc Trop Med Hyg. 2009;103:45–51. 18951599 10.1016/j.trstmh.2008.08.018

[36] Vinkeles Melchers NV, van Dam GJ, Shaproski D, Kahama AI, Brienen EA, Vennervald BJ, et al. Diagnostic performance of Schistosoma real-time PCR in urine samples from Kenyan children infected with Schistosoma haematobium: day-to-day variation and follow-up after praziquantel treatment. PLoS Negl Trop Dis. 2014;8:e2807. 24743389 10.1371/journal.pntd.0002807 PMC3990496

[37] Obeng BB, Aryeetey YA, de Dood CJ, Amoah AS, Larbi IA, Deelder AM, et al. Application of a circulating-cathodic-antigen (CCA) strip test and real-time PCR, in comparison with microscopy, for the detection of Schistosoma haematobium in urine samples from Ghana. Ann Trop Med Parasitol. 2008;102:625–33. 18817603 10.1179/136485908X337490

[38] Hauser J, Lenk G, Hansson J, Beck O, Stemme G, Roxhed N. High-yield passive plasma filtration from human finger prick blood. Anal Chem. 2018;90:13393–9. 30379058 10.1021/acs.analchem.8b03175

[39] Cnops L, Soentjens P, Clerinx J, Van Esbroeck M. A Schistosoma haematobium-specific real-time PCR for diagnosis of urogenital schistosomiasis in serum samples of international travelers and migrants. PLoS Negl Trop Dis. 2013;7:e2413. 24009791 10.1371/journal.pntd.0002413 PMC3757062

[40] Fuss A, Mazigo HD, Mueller A. Evaluation of serum-based real-time PCR to detect Schistosoma mansoni infection before and after treatment. Infect Dis Poverty. 2020;9:74. 32571433 10.1186/s40249-020-00698-z PMC7309987

[41] Wichmann D, Panning M, Quack T, Kramme S, Burchard GD, Grevelding C, et al. Diagnosing schistosomiasis by detection of cell-free parasite DNA in human plasma. PLoS Negl Trop Dis. 2009;3:e422. 19381285 10.1371/journal.pntd.0000422 PMC2667260

[42] Wichmann D, Poppert S, Von Thien H, Clerinx J, Dieckmann S, Jensenius M, et al. Prospective European-wide multicentre study on a blood based real-time PCR for the diagnosis of acute schistosomiasis. BMC Infect Dis. 2013;13:55. 23363565 10.1186/1471-2334-13-55 PMC3563621

[43] Guegan H, Fillaux J, Charpentier E, Robert-Gangneux F, Chauvin P, Guemas E, et al. Real-time PCR for diagnosis of imported schistosomiasis. PLoS Negl Trop Dis. 2019;13:e0007711. 31509538 10.1371/journal.pntd.0007711 PMC6756557

[44] Frickmann H, Lunardon LM, Hahn A, Loderstadt U, Lindner AK, Becker SL, et al. Evaluation of a duplex real-time PCR in human serum for simultaneous detection and differentiation of Schistosoma mansoni and Schistosoma haematobium infections - cross-sectional study. Travel Med Infect Dis. 2021;41:102035. 33775915 10.1016/j.tmaid.2021.102035

[45] Ouedraogo M, Hey JC, Hilt S, Rodriguez Fernandez V, Winter D, Razafindrakoto R, et al. Comparative evaluation of plasma biomarkers of Schistosoma haematobium infection in endemic populations from Burkina Faso. PLoS Negl Trop Dis. 2024;18:e0012104. 39292709 10.1371/journal.pntd.0012104 PMC11441675

[46] Berry SPD, Honkpehedji YJ, Ludwig E, Mahmoudou S, Prodjinotho UF, Adamou R, et al. Impact of helminth infections during pregnancy on maternal and newborn Vitamin D and on birth outcomes. Sci Rep. 2024;14:14845. 38937587 10.1038/s41598-024-65232-9 PMC11211496

[47] Honkpehedji YJ, Adegbite BR, Zinsou JF, Dejon-Agobe JC, Edoa JR, Zoleko Manego R, et al. Association of low birth weight and polyparasitic infection during pregnancy in Lambarene, Gabon. Trop Med Int Health. 2021;26:973–81. 33860600 10.1111/tmi.13591

[48] Dejon-Agobe JC, Edoa JR, Adegnika AA, Grobusch MP. Schistosomiasis in Gabon from 2000 to 2021 - a review. Acta Trop. 2022;228:106317. 35051384 10.1016/j.actatropica.2022.106317

[49] Meulah B, Oyibo P, Hoekstra PT, Moure PAN, Maloum MN, Laclong-Lontchi RA, et al. Validation of artificial intelligence-based digital microscopy for automated detection of Schistosoma haematobium eggs in urine in Gabon. PLoS Negl Trop Dis. 2024;18:e0011967. 38394298 10.1371/journal.pntd.0011967 PMC10917302

[50] Kouna LC, Oyegue-Liabagui SL, Atiga CN, Mbani Mpega Ntigui CN, Imboumy-Limoukou RK, Biteghe Bi Essone JC, et al. molecular detection of urogenital schistosomiasis in community level in semi-rural areas in South-East Gabon. Diagnostics (Basel). 2025;15:1052. 40361870 10.3390/diagnostics15091052 PMC12071237

[51] Colombe S, Lee MH, Masikini PJ, van Lieshout L, de Dood CJ, Hoekstra PT, et al. Decreased sensitivity of Schistosoma sp. egg microscopy in women and HIV-infected individuals. Am J Trop Med Hyg. 2018;98:1159–64. 29405114 10.4269/ajtmh.17-0790 PMC5928829

[52] Tweyongyere R, Mawa PA, Kihembo M, Jones FM, Webb EL, Cose S, et al. Effect of praziquantel treatment of Schistosoma mansoni during pregnancy on immune responses to schistosome antigens among the offspring: results of a randomised, placebo-controlled trial. BMC Infect Dis. 2011;11:234. 21888656 10.1186/1471-2334-11-234 PMC3176493

[53] Aguree S, Gernand AD. Plasma volume expansion across healthy pregnancy: a systematic review and meta-analysis of longitudinal studies. BMC Pregnancy Childbirth. 2019;19:508. 31856759 10.1186/s12884-019-2619-6 PMC6924087

[54] Honkpehedji YJ, Adegnika AA, Dejon-Agobe JC, Zinsou JF, Mba RB, Gerstenberg J, et al. Prospective, observational study to assess the performance of CAA measurement as a diagnostic tool for the detection of Schistosoma haematobium infections in pregnant women and their child in Lambarene, Gabon: study protocol of the freeBILy clinical trial in Gabon. BMC Infect Dis. 2020;20:718. 32993559 10.1186/s12879-020-05445-1 PMC7523491

[55] Honkpehedji YJ, Jean-Claude D-A, Jeannot Fréjus Z, Romuald Beh M, Jacob G, Ronald E, et al. PA-166 Circulating anodic antigen (CAA) detection in pregnant women and their child during Schistosoma haematobium infections in Lambaréné, Gabon. BMJ Glob Health. 2023. 10.1136/bmjgh-2023-EDC.110.

[56] Adam I, Al-Habardi NA, Al-Wutayd O, Khamis AH. Prevalence of schistosomiasis and its association with anemia among pregnant women: a systematic review and meta-analysis. Parasit Vectors. 2021;14:133. 33653391 10.1186/s13071-021-04642-4 PMC7923606

[57] Bengu MD, Dorsamy V, Moodley J. Schistosomiasis infections in South African pregnant women: a review. S Afr J Infect Dis. 2020;35:171. 39380900 10.4102/sajid.v35i1.171 PMC11459292

[58] Mombo-Ngoma G, Honkpehedji J, Basra A, Mackanga JR, Zoleko RM, Zinsou J, et al. Urogenital schistosomiasis during pregnancy is associated with low birth weight delivery: analysis of a prospective cohort of pregnant women and their offspring in Gabon. Int J Parasitol. 2017;47:69–74. 28003151 10.1016/j.ijpara.2016.11.001

[59] Ndiour CN, Senghor B, Thiam O, Niang S, Wotodjo AN, Faye BT, et al. Prevalence and associated factors of schistosomiasis among pregnant women in northern Senegal. BMC Infect Dis. 2024;24:682. 38982383 10.1186/s12879-024-09443-5 PMC11232235

