# Supplementary material for: Diagnostic accuracy of plasma cell-free DNA qPCR for Schistosoma haematobium assessed by Bayesian latent class analysis in a cohort of pregnant women from Lambaréné, Gabon
Source: Infect Dis Poverty. 2026 May 9;15:50. doi: 10.1186/s40249-026-01447-4 (PMC13156850; doi:10.1186/s40249-026-01447-4)
Supplement: Supplementary file 1 — Supplementary material 1. [file 40249_2026_1447_MOESM1_ESM.docx]

**Supplementary Data**


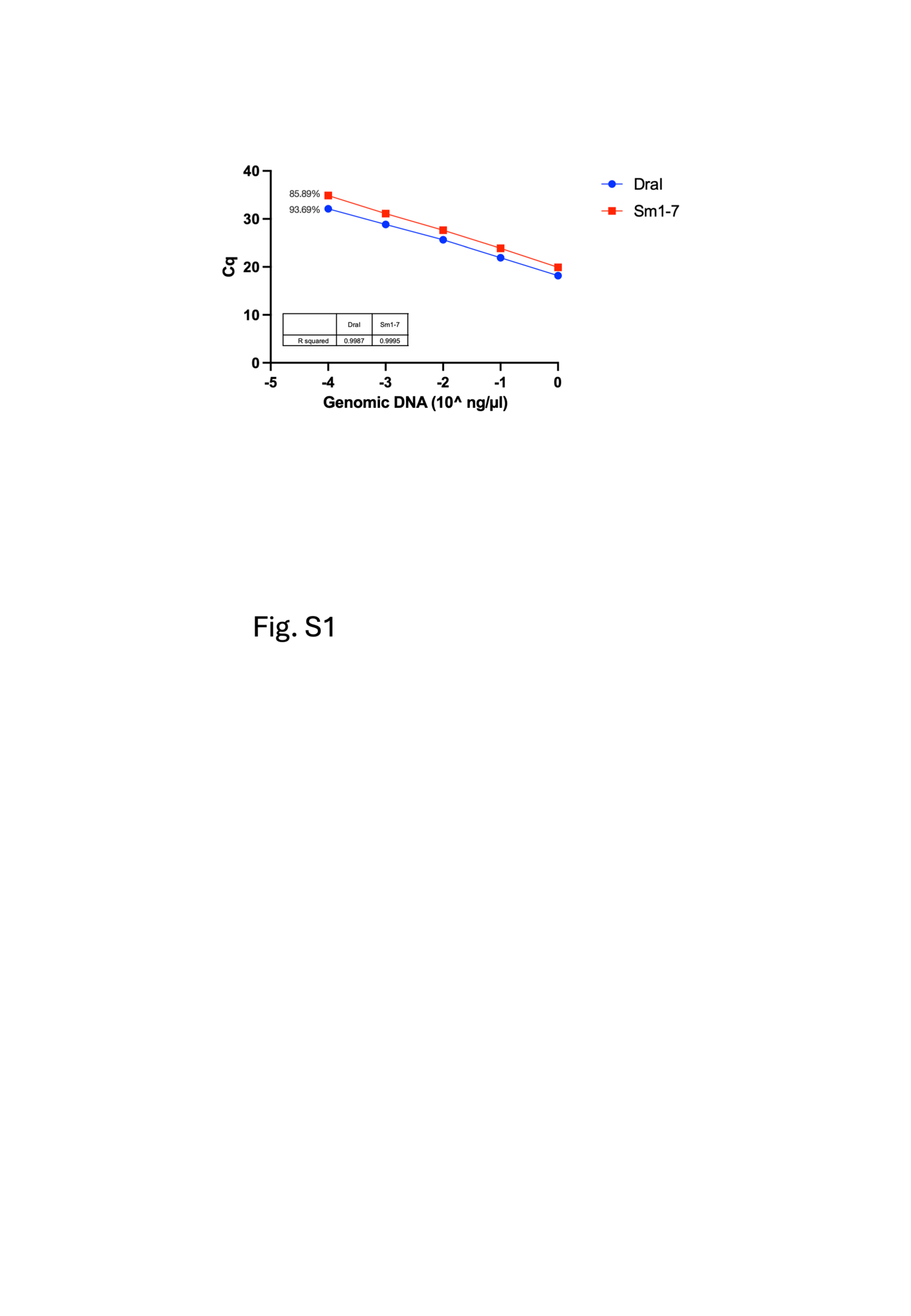


Supplementary Fig. 1. Validation of the Sm1-7 and Dra1 qPCR assays for *S. mansoni* and *S. haematobium*, respectively*.* In a correlation curve of Ct vs. genomic DNA concentration, Dra1 exhibited the most linear and most sensitive performance amongst the 2 assays.


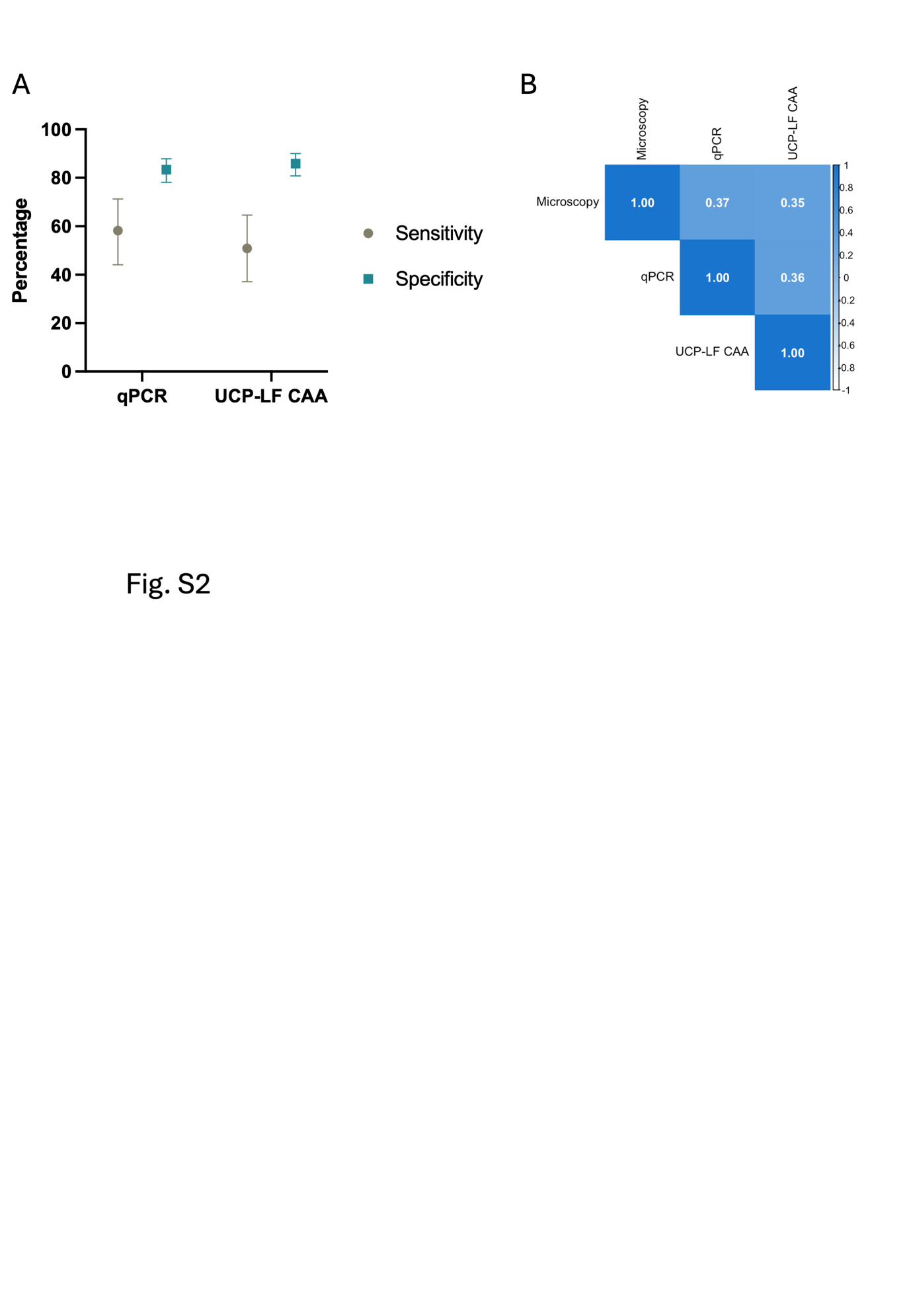


Supplementary Fig. 2. A) Sensitivity and specificity (estimate and 95% CI) of each diagnostic test as compared to urine filtration microscopy as a reference standard. B) Matrix Cohen’s kappa coefficient for each test pair indicating fair to moderate agreement.

Table S1. qPCR performance as compared to a composite reference standard of urine filtration microscopy and UCP-LF CAA.

| Test | Sensitivity | Specificity | Accuracy | Youden | Cohen’s kappa |
| --- | --- | --- | --- | --- | --- |
| qPCR | 52.8.% (41.9–63.5) | 87.9% (82.7–92.0) | 77.4% (72.2–82.0) | 0.407 | 0.431 |
| UCP-LF CAA | 69.7% (59.0–79.0) | 100.0% (98.2–100.0) | 90.9% (87.0–93.9) | 0.697 | 0.763 |
| UF microscopy | 61.8.% (50.9–71.9) | 100.0% (98.2–100.0) | 88.5% (84.3–91.9) | 0.618 | 0.693 |

Accuracy = proportion of all correctly classified individuals

Youden’s J = combined measure of sensitivity and specificity, higher is better.

Table S2. Comparison of diagnostic metrics as compared to urine filtration microscopy.

| Test | Sensitivity | Specificity | Accuracy | Youden |
| --- | --- | --- | --- | --- |
| qPCR | 58.2% (44.1–71.3) | 83.4% (78.1–87.9) | 78.7% (73.6–83.2) | 0.42 |
| UCP-LF CAA | 50.9% (37.1–64.6) | 85.9% (80.8–90.0) | 79.4% (74.3–83.9) | 0.37 |

Accuracy = proportion of all correctly classified individuals

Youden = combined measure of sensitivity and specificity, higher is better.

Table S3. Diagnostic metrics calculated using BLCA.

Model 1: Informative priors with truncation. Model 2: Informative priors, no truncation. Model 3: flat priors with truncation.

|  | Prevalence | Test | Sensitivity (95% CrI) | Specificity (95% CrI) | Accuracy | Youden |
| --- | --- | --- | --- | --- | --- | --- |
| Model 1 | 22.5% | qPCR | 73.9% (57.0–90.7) | 89.3% (83.4–94.9) | 85.8% | 0.632 |
|  |  | UCP-LF CAA | 65.5% (48.7–83.0) | 91.3% (85.9–96.0) | 85.5% | 0.568 |
|  |  | Microscopy | 60.7% (44.6–78.2) | 91.9% (87.2–95.9) | 84.9% | 0.526 |
| Model 2 | 22.5% | qPCR | 74.0% (57.2–90.8) | 89.3% (83.3–95.1) | 85.8% | 0.632 |
|  |  | UCP-LF CAA | 65.7% (49.0–82.8) | 91.3% (86.1–96.1) | 85.6% | 0.570 |
|  |  | Microscopy | 60.5% (43.8–78.4) | 91.9% (87.2–96.0) | 84.8% | 0.524 |
| Model 3 | 27.1% | qPCR | 68.6% (51.8–86.6) | 91.6% (84.9–98.4) | 85.3% | 0.602 |
|  |  | UCP-LF CAA | 60.0% (44.5–78.0) | 92.9% (87.0–98.6) | 84.0% | 0.529 |
|  |  | Microscopy | 57.1% (42.2–75.6) | 94.9% (89.6–99.3) | 84.7% | 0.520 |

**
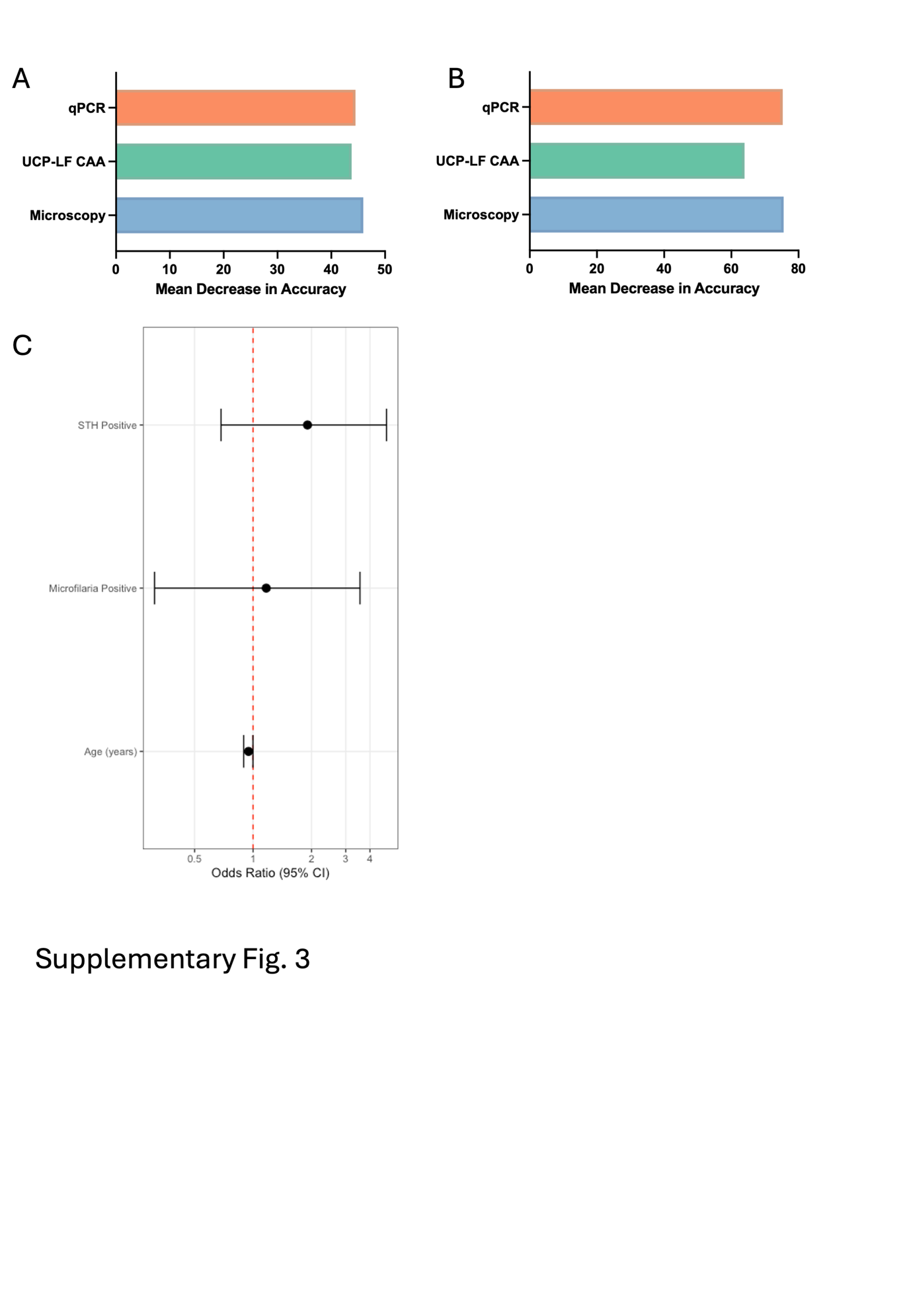
**

Supplementary Fig. 3. A and B) Importance of diagnostic tests to LCA-defined schistosomiasis status using binary (A) or continuous (B) test results, identified using a Random Forest classifier. Variable importance is ranked using the mean decrease in accuracy. C) Odds ratio and 95% CI from multivariate logistic regression to identify association between co-infections or age, and schistosomiasis-status. This identified trends towards co-infections and a younger tendency for schistosomiasis-positive cases, though no significant co-variates.


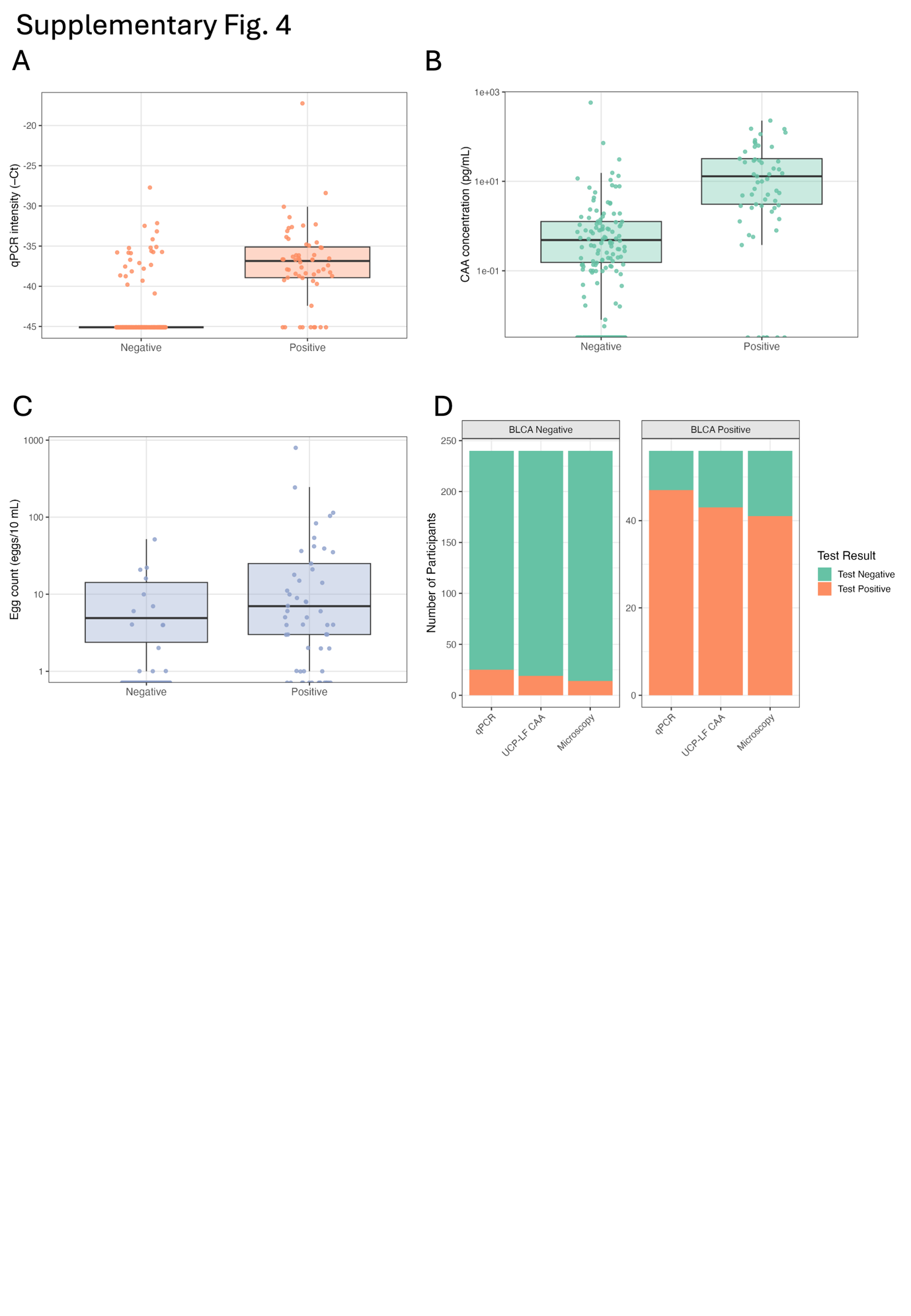


Supplementary Fig. 4. A-C. Mean and IQR for each diagnostic test portraying each sample tested, plotting LCA-derived schistosomiasis-status vs. quantitative measurement of diagnostic value. D) Comparison of the positivity of each diagnostic test as compared to BLCA positivity (Model 2).

**Supplementary methods**

**Covariate analysis**

We explored associations between LCA-inferred infection status and age, microfilaria positivity, and soil-transmitted helminth (STH) coinfection. Logistic regression models were used to estimate odds ratios and 95% confidence intervals for each covariate.

**Random forest classification**

We applied Random Forest models to identify which diagnostic tests best predicted LCA-inferred infection status. Models were constructed using either binary or continuous test variables. Feature importance was extracted via mean decrease in accuracy and visualized to rank diagnostic relevance. All modeling was conducted using the randomForest and caret packages in R.
